# Meditation modalities for ADHD in minority pediatric populations in the USA: a scoping review

**DOI:** 10.34172/hpp.42837

**Published:** 2024-07-29

**Authors:** Shahrzad Bazargan-Hejazi, Christopher Martin, Bellamy Hall, Jeneane Hamideh, Miranda Lam, Antonia Osuna-Garcia, Darlene Parker-Kelly, Derek O. Pipolo, Myra Usmani, Stacey A. Teruya

**Affiliations:** ^1^Charles R. Drew University of Medicine and Science, 1731 E. 120th St., Los Angeles, CA 90059, USA; ^2^David Geffen School of Medicine, University of California at Los Angeles, 1731 E. 120th St., Los Angeles, CA 90059, USA; ^3^Charles R. Drew University of Medicine and Science, Research Library, 1731 E. 120th St., Los Angeles, CA 90059, USA; ^4^University of California at Los Angeles, Biomedical Library, 2-077 Ctr For Health Sciences, Los Angeles, CA 90095, USA; ^5^Department of Neurological Surgery, Trauma and Emergency Hospital “Dr. Federico Abete”, Buenos Aires, Argentina; ^6^University of California at Riverside, College of Natural and Agricultural Sciences, Riverside, CA 92507, USA

**Keywords:** Health disparity, Minority and vulnerable, Attention deficit disorder, Attention deficit disorder with hyperactivity, Mediation, Mindfulness

## Abstract

**Background:**

Roughly 10% of children aged 3 -17 in the USA are diagnosed with attention-deficit hyperactivity disorder (ADHD), and minorities are less likely to initiate common pharmacologic treatment. We conducted a review of the literature to examine meditation as a safe, effective, and low-cost alternative.

**Methods:**

We searched PubMed and other journals using "meditation," "mindfulness," "minority," related keywords, and relevant MeSH terms. Eligible studies involved racial/ethnic minorities in the USA, reported quantitative psychosocial outcomes, and were published in a peer-reviewed, English-language journal.

**Results:**

Out of 119 "hits," 111 were eliminated as duplicates or were not relevant. A full-text review of the remaining eight revealed that none fully met our eligibility criteria. Besides the obvious lack of studies, those reviewed reported incomplete demographic and clinical data. They also employed different and inconsistent research methodologies, interventions and modalities, and statistical analyses. This hindered understanding exactly which populations may benefit from meditation, and for which specific symptoms.

**Conclusion:**

We recommend a socio-ecological model in examining intervention modalities, especially in the context of intrapersonal, interpersonal, organizational, environmental, and policy domains. We also suggest the possible inclusion of research older than 10 years, conducted outside of the USA, on minority and non-minority populations, for supplementary and confirmatory data. We advocate for consistency in study design and data collection, which would help align research conducted in different countries. Searches should also include variations of meditation such as "mindfulness" and "guided imagery," and associated symptoms and comorbidities of ADHD, including "learning disorder" and "behavioral problems."

## Introduction

 Attention-deficit hyperactivity disorder (ADHD) is a neurodevelopmental condition that interferes with functioning and development in children, characterized by persistent patterns of inattention and hyperactivity-impulsivity.^1^ A 2009-2017 National Health Interview Survey found that nearly 10% of children aged 3-17 suffered from ADHD,^2^ with a slightly lower prevalence (9.85%) among non-Hispanic Black children.^3^ In the general population, ADHD co-occurs with anxiety disorder in 25% of children, and in 30%-40% of those who are clinically-referred. ADHD is present in 30 - 50% of children diagnosed with oppositional defiant disorder (ODD) and conduct disorder (CD).^4^ Children with CD also have an increased risk of bipolar disorder.^5-9^

 There is evidence of an ethnic disparity in the diagnosis and treatment of ADHD among minority children.^10-13^ Parental attitudes toward mental health issues and medication use, and the underutilization of mental health services by minorities, appear to be contributing factors.^14^ Paradoxically, minority children may be over-diagnosed and over-treated for ADHD. Their abilities and behaviors may more often be subjectively viewed as problematic and atypical,^15,16^ requiring intervention and treatment. Possibly as a result, there is a disproportionately high percentage of minority children in special education.^17,18^

 It is important to note that nearly 20% of patients do not respond well to commonly prescribed ADHD medication. Adverse side effects include mood, anxiety and sleep disorders, and substance misuse.^19^ It is therefore not surprising that over 70% of ADHD patients choose non-pharmacological interventions,^20^ of which meditation may be effective.^21^ Functional magnetic resonance imaging (fMRI) of the brains of ADHD patients has shown that meditation can counter hypo-activation in key regions including the anterior cingulate cortex, prefrontal cortices, parietal cortices, and the basal ganglia.^22^ Meditation may also influence the cerebral parenchyma, increasing gray matter thickness in many of these regions.^22-25^ Meditation may also be associated with neural and metabolic changes and responses that can ameliorate ADHD and associated comorbidities,^26^ and with the inhibition of the sympathetic nervous system.^27^

 Credible and rigorous research that supports meditation-based interventions as comparable to, or more effective than, current standard-of-care could influence best practices in both pediatrics and psychiatry. The result may be a safe, effective, low-cost and accessible alternative to pharmacological interventions that would be especially beneficial for children from racial and ethnic minorities who suffer from ADHD.

###  Research aims

 The primary aim of this study was to assess the efficacy and potential benefits of meditation as a treatment modality for minority, pediatric ADHD patients. Our objectives included reporting and discussing findings in the literature, identifying gaps and limitations in existing research, and to offer recommendations that would guide and shape future study. Through these, we sought to contribute to a better understanding of the role of meditation in addressing ADHD among pediatric patients from diverse racial and ethnic backgrounds.

## Material and Methods

###  Study design and database search

 We followed Preferred Reporting Items for Systematic Reviews and Meta-Analyses (PRISMA) guidelines^28^ in conducting our search of PubMed, Web of Science, PsycINFO, Embase, and Cochrane Review for relevant articles published between December, 2012 and December 2023, inclusive. We employed a large list of keywords that included “meditation,” “mindfulness,” and “attention deficit disorder with hyperactivity,” along with relevant MeSH terms (Table S1).

###  Search criteria

 Inclusion criteria were established using a framework of* population, intervention, a control or comparison group, *and* outcomes of interest* (PICO).^29^ Eligible studies considered minority ADHD pediatric patients, age 17 and under, who were diagnosed with the following International Classification of Diseases (ICD) codes:

- F90.0 Attention-deficit hyperactivity disorder, predominantly inattentive type.
- F90.1 Attention-deficit hyperactivity disorder, predominantly hyperactive type.
- F90.2 Attention-deficit hyperactivity disorder, combined type.
- F90.8 Attention-deficit hyperactivity disorder, other type.
- F90.9 Attention-deficit hyperactivity disorder, unspecified type.

We included ADHD populations with comorbidities such as anxiety, depression, CD, and substance use disorder.

###  Eligibility criteria

 Eligible studies used meditation modalities to treat ADHD in minority pediatric populations in the USA. Studies that involved control or comparison groups used medication and/or current standard of care. Participants may also have received no intervention or treatment, or received placebos. Eligible studies were peer-reviewed, involved participants in the USA, and were published between December 2012 and December 2023, inclusive. Study measurements must have included (1) reduction (if any) of core symptoms, (2) global functioning/quality of life, and (3) social functioning. Functioning/quality of life and social function must have been measured through validated scales.

###  Study selection 

**Figure 1:**
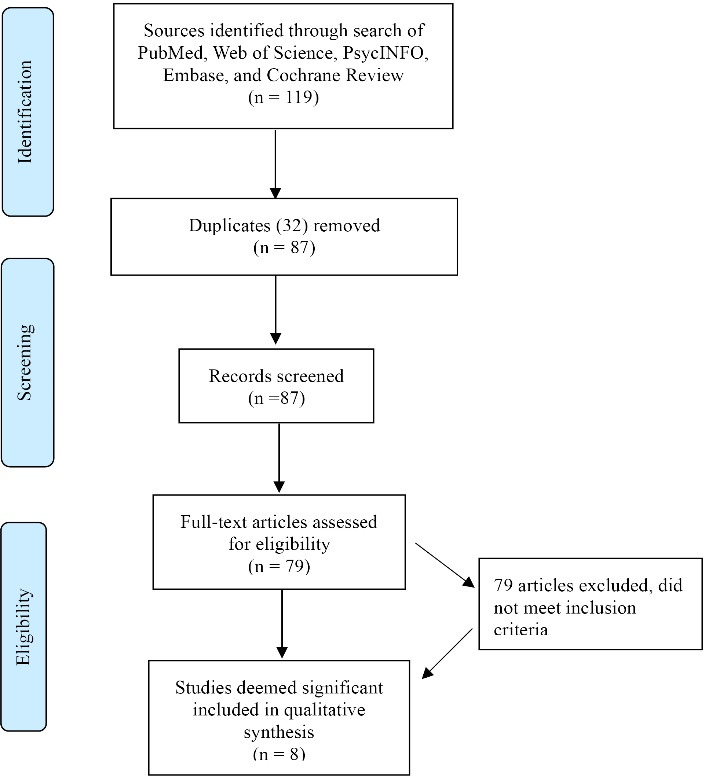
PRISMA Flow chart of source selection. Our initial search yielded 119 journal articles and manuscripts. Of these, none met our strict inclusion criteria. However, eight of these reported improvement through the use of meditation and related modalities

 We used *Covidence*, a systematic review platform (Veritas Health Innovation, Melbourne, Australia; http://www.covidence.org/) to screen potential studies in several stages for inclusion in our final review (Figure 1). In the first stage, two study members independently reviewed the titles and summaries of “hits” found in our initial search for terms and keywords relevant to our research aims. In the second stage, the abstracts of relevant studies were reviewed to confirm their potential inclusion. In the third stage, a full-text review was conducted to determine whether the article met eligibility criteria and should be included in our final analysis. To demonstrate reliability in our review process, we examined inter-rater consistency, and resolved discrepancies through discussion and consensus.

 We limited our search to studies published in peer-reviewed journals between December 2012 and December 2023, inclusive. This was done primarily because meditation-based interventions are seen to have been increasingly utilized and promoted as an alternative or complementary treatment for ADHD only within the past decade. However, it is possible that earlier studies may have also proved relevant and useful.

 We searched using “meditation” as the main intervention in research that involved participants 17 years of age or younger diagnosed specifically with ADHD. We considered only those studies published in peer-reviewed journals between December 2012 and December 2023, inclusive. This was done primarily because meditation-based interventions are seen to have been increasingly utilized and promoted as an alternative or complementary treatment for ADHD only within the past decade.^30-32^

## Results

 Our search of the literature yielded 119 “hits” with 32 duplicates. We screened the titles and abstracts of the remaining 87 and eliminated 79 studies based on relevance and inclusion criteria. Through a full-text review of the remaining eight articles, we found that none fully met our eligibility criteria (Figure 1). However, these articles did report significant, although inconsistent, equivocal, and possibly subjective positive improvement through the use of meditation and related modalities. Moreover, cultural, socioeconomic, and clinical details of participants in these eight studies were often inconsistent and incomplete. This hindered identifying which interventions were effective for specific populations. Methodological differences across these studies also made it difficult to discern the most effective modalities and options for specific conditions and symptoms of ADHD.

###  Studies conducted outside of the USA

 An individualized intervention conducted in Iran, for example, found perceptual-motor training and mindfulness associated with a statistically significant improvement among children with ADHD. However, this was found only in “certain subgroups (indicators) of attention deficit hyperactivity networks.”^33^ A family-centered program in Holland^34^ taught youths with ADHD mindfulness techniques, while their parents were trained in mindful parenting. In this qualitative study, many parents reported improvement in ADHD symptoms, although these were diverse and largely inconsistent.

###  Studies conducted in the USA

 Several studies conducted in the USA^35-39^ did not meet our eligibility criteria, as they did not specifically examine ADHD. However, these reported improvement in associated symptoms such as impulsivity, behavioral dysregulation, and attentional capacity difficulties associated with reactive aggression. One of these studies involved “Coping Power” (CP),^35^ an evidence-based intervention for youth with disruptive behavior problems in suburban Alabama. It was compared to “Mindful Coping Power” (MCP), a variation of CP that incorporates elements of mindfulness. Children received their respective intervention during the school week in 45-minute sessions, while parent group sessions occurred once a week. The MCP intervention was associated with a statistically significant improvement in self-reported dysregulation, inhibitory control and breath awareness. However, no parent-reported improvement was statistically significant.

 Another school-based mindfulness intervention^39^ involved third- through sixth-grade public charter school students, and their teachers. Both groups learned mindfulness practices together for 30-40 minutes per week over ten weeks. Ninety-three percent of the participants were Hispanic/Latino or African American. This intervention was associated with significantly higher cooperation levels, and significantly lower hyperactivity/inattention in the children, as reported by teachers. Males had significantly lower hyperactivity/inattention scores than those in the control group; this effect was not seen in females.

 “Move Into Learning” is an eight-week mindfulness-based educational intervention consisting of yoga, music, and visual arts that was implemented among urban, underserved third graders.^36^ Although it did not consider ADHD *per se*, the researchers found a statistically significant improvement in hyperactivity, and in cognitive ability and inattentiveness. This improvement persisted through post-intervention and a two-month follow-up assessment. Interviews with the teachers also revealed their positive perceptions regarding the feasibility and usefulness of the intervention.

 A study of high school students in a 12-week intervention program that employed yoga and meditation sessions^37^ found significant improvement in self-reported measures of attention and hyperactivity. Teachers also reported that students who completed the intervention were more attentive in class and presented better posture. A review^38^ of school-based, mind-body practices and interventions focused on children aged 2-18. It included yoga, meditation, mindfulness-based stress reduction, and relaxation training techniques. Although there was improvement in specific ADHD-related symptoms, it was unclear exactly which interventions and practices were most effective.

 Other studies^40-42^ suggest that “acceptance and commitment” therapy, which relies heavily on mindfulness practices, may be effective for Black adolescents with comorbid ADHD, learning disorders, and behavior problems. Significant improvement was also seen in self-reported measures of attention and hyperactivity.

## Discussion

 Although we found no articles that met our rigorous eligibility criteria, our full-text review yielded findings and information which can help guide and inform future research. Articles examined in this stage often revealed incomplete cultural, socioeconomic, and clinical details of participants, perhaps as a function of study design and data collection. This hindered identifying which interventions may be the most effective for specific racial/ethnic and socioeconomic populations and (sub)groups. In addition, because of the substantial differences in research methodologies, modalities, and statistical analyses, it was difficult to determine which interventions and programs were the most effective in treating specific components and related symptoms of ADHD in minority children. Consistency in study design and data collection would also help align research conducted in the USA and in different countries.

 A variety of meditation modalities have been applied in treating ADHD and related symptoms in children, with different levels and measures of success. To analyze, assess and organize these different approaches and modalities, we employed a unifying socio-ecological model^43^ of child development and environmental context relevant in promoting health-related behavioral change at home, in schools, and in other settings. We considered meditation modalities, interventions and programs as they may operate in and across intrapersonal, interpersonal, organizational, environmental, and policy levels of this framework.^44^

 At the intrapersonal level, practices like mindfulness and yoga appear to empower children in enhancing self-regulation and attention. Family-based mindfulness programs at the interpersonal level improve communication and coping skills for both children, and for their families. The informed perspectives and participation of parents and educators may also operate at this level. Mindfulness-based classroom interventions are seen to act within the organizational domain. These allow administrators to adapt educational practices to better serve children with ADHD. Within environmental and policy domains, factors such as better access to resources and mental health services may significantly mitigate ADHD symptomatology.

## Limitations

 Our study focused exclusively on interventions for ADHD in racial and ethnic minorities within the USA. There may exist a substantial body of research involving non-minority children, or studies that involve various and diverse racial and ethnic groups. Excluding non-minority populations could potentially lead to an incomplete understanding of meditation interventions for ADHD.

 We searched using “meditation” as the main intervention. A search of related modalities, variations, practices, and techniques such as “transcendentalism,” “mantra,” “yoga,” “chakra,” and “guided imagery” may have produced a larger number of “hits,” and could have yielded additional articles for consideration. Similarly, we considered only those studies that involved participants diagnosed specifically with ADHD. By expanding our search to include common ADHD comorbidities such as behavior or conduct problems, learning disorders, anxiety and depression, we may have increased the number of studies for potential inclusion.

 We also recognize that valuable insight may exist in studies conducted outside of the USA. A more global perspective on ADHD interventions for children could offer supplementary or supporting material and contribute to the comprehensiveness of our findings. Future research may therefore incorporate a more diverse range of study populations and consider international perspectives to achieve a more holistic understanding of the efficacy of meditation in treating ADHD.

## Conclusions and Recommendations

 Our search of the literature returned no studies that met our strict eligibility criteria. This underscores the need for more comprehensive and standardized research in meditation and mindfulness interventions for ADHD in minority children. Cultural, socioeconomic, and clinical details of participants were often inconsistent and incomplete, which hindered identifying interventions that were effective for specific populations. In addition, methodological differences across studies made it difficult to discern the most effective modalities and options for specific conditions and symptoms of ADHD.

 Despite these limitations, articles examined in our review process provided valuable insight into diverse approaches. By utilizing a unifying, socio-ecological model to analyze meditation modalities, we were able to discern their impact in different domains of child development and environmental contexts. Mindfulness and yoga interventions showed promise at the intrapersonal level, fostering self-regulation and attention in children. Family-based programs appeared beneficial at the interpersonal level, by enhancing communication and coping skills. Classroom interventions operated within the organizational domain, empowering administrators in adopting better educational practices for children with ADHD. Environmental and policy factors, such as improved access to resources and mental health services, were identified as crucial components in mitigating ADHD symptomatology.

 While no studies fully met our eligibility criteria, findings from this review help lay a foundation for future research. Understanding the effects of meditation across diverse populations and settings is essential in developing safe, targeted and effective interventions for children with ADHD.

## Acknowledgements

 Heartfelt thanks to Kaveh Dehghan, MBA, for his exceptional support and wisdom in finalizing this manuscript; his assistance has been invaluable.

## Competing Interests

 None.

## Ethical Approval

 Not applicable.

## Supplementary Files

 Table S1. PubMed, Web of Science, PsychInfo, Embase, and Cochrane Review keywords, MeSH terms, and search results
