# Supplementary material for: Meditation modalities for ADHD in minority pediatric populations in the USA: a scoping review
Source: Health Promot Perspect. 2024 Jul 29;14(2):91–6. doi: 10.34172/hpp.42837 (PMC11403344; doi:10.34172/hpp.42837)
Supplement: Supplementary file 1 — Table S1. PubMed, Web of Science, PsychInfo, Embase, and Cochrane Review keywords, MeSH terms, and search results [file hpp-14-91-s001.pdf]

Bazargan-Hejazi et al, **Health Promotion Perspectives,** 2024, 14(2), 91-S1
doi: 10.34172/hpp.42837
https://hpp.tbzmed.ac.ir

Table S1 . PubMed, Web of Science, PsychInfo, Embase, and Cochrane Review keywords, MeSH terms, and search results

**1. PubMed search with minority added.**		
**Concept**	**Controlled Vocab**	**Keywords**
Minority/underrepres ented	"Health Disparity, Minority and Vulnerable Populations"[Mesh] OR "Minority Health"[Mesh:NoExp] OR "Minority Groups"[Mesh:NoExp] OR "Ethnic and Racial Minorities"[Mesh:NoExp]	underserved[Title/Abstract] OR minority[Title/Abstract] OR minorities[Title/Abstract] OR homeless*[Title/Abstract] OR impaired[Title/Abstract] OR uninsured[Title/Abstract] OR underinsured[Title/Abstract] OR african*[Title/Abstract] OR black[Title/Abstract] OR blacks[Title/Abstract] OR "native american"[Title/Abstract] OR "native americans"[Title/Abstract] OR gypsy[Title/Abstract] OR gypsies[Title/Abstract] OR hispanic[Title/Abstract] OR hispanics[Title/Abstract] OR latino[Title/Abstract] OR latinos[Title/Abstract] OR Latina[Title/Abstract] OR latinas[Title/Abstract] OR chicano[Title/Abstract] OR chicanos[Title/Abstract] OR "mexican american"[Title/Abstract] OR "mexican americans"[Title/Abstract] OR mexican-american*[Title/Abstract] OR "puerto rican"[Title/Abstract] OR "puerto ricans"[Title/Abstract] OR jamaican[Title/Abstract] OR jamaicans[Title/Abstract] OR immigrant[Title/Abstract] OR immigrants[Title/Abstract] OR refugee[Title/Abstract] OR refugees[Title/Abstract] OR transients[Title/Abstract] OR migrant[Title/Abstract] OR migrants[Title/Abstract]
Children	"Child"[Mesh:NoExp] OR "Pediatrics"[Mesh:NoExp] OR "Adolescent"[Mesh:NoExp]	Child*[Title/Abstract] OR pediatric*[Title/Abstract] OR adolescen*[Title/Abstract] OR "young adult"[Title/Abstract] OR youth[Title/Abstract]
ADHD	"Attention Deficit Disorder with Hyperactivity"[Mesh:NoExp] OR "Attention Deficit and Disruptive Behavior Disorders"[Mesh:NoExp] OR "Attention Deficit Disorder with Hyperactivity/therapy"[Mesh:NoExp] OR "Hyperkinesis"[Mesh:NoExp]	"Attention Deficit Disorder"[Title/Abstract] OR ADHD[Title/Abstract] OR "Attention Deficit Disorder with Hyperactivity"[Title/Abstract] OR ADD[Title/Abstract] OR hyperactiv*[Title/Abstract] OR hyperkinesis[Title/Abstract] OR inattention[Title/Abstract] OR impulsive*[Title/Abstract] OR overactive*[Title/Abstract] OR externaliz*[Title/Abstract]
Meditation	"Meditation"[Mesh:NoExp] OR "Mindfulness"[Mesh:NoExp]	Meditat*[Title/Abstract] OR mindful*[Title/Abstract] OR transcendental[Title/Abstract] OR vipassana[Title/Abstract] OR “loving kindness”[Title/Abstract] OR “mindful-self compassion”[Title/Abstract] OR yoga[Title/Abstract] OR zen[Title/Abstract] OR sudarshan[Title/Abstract] OR “chi kung”[Title/Abstract] OR qigong[Title/Abstract] OR kirtan[Title/Abstract] OR pranayama[Title/Abstract] OR “acceptance commitment therapy”[Title/Abstract]
Results	All concepts AND-ed: 11 results<br>Date limit 2012-2023: 10 results<br>Searched on 12/31/23	Relevant references:<br>29991532[PMID] – not included<br>28547119[PMID] – not included
**2. Embase**		
**Concept**	**Controlled Vocab**	**Keywords**
Minority	'vulnerable population'/de OR 'disadvantaged population'/de OR 'minority health'/de OR 'minority group'/de OR 'ethnic group'/de	underserved:ti,ab OR minority:ti,ab OR minorities:ti,ab OR homeless*:ti,ab OR impaired:ti,ab OR uninsured:ti,ab OR underinsured:ti,ab OR african*:ti,ab OR black:ti,ab OR blacks:ti,ab OR ‘native american’:ti,ab OR ‘native americans’:ti,ab OR gypsy:ti,ab OR gypsies:ti,ab OR hispanic:ti,ab OR hispanics:ti,ab OR latino:ti,ab OR latinos:ti,ab OR latina:ti,ab OR latinas:ti,ab OR chicano:ti,ab OR chicanos:ti,ab OR ‘mexican american’:ti,ab OR ‘mexican americans’:ti,ab OR mexican-american*:ti,ab OR ‘puerto rican’:ti,ab OR ‘puerto ricans’:ti,ab OR jamaican:ti,ab OR jamaicans:ti,ab OR immigrant:ti,ab OR immigrants:ti,ab OR refugee:ti,ab OR refugees:ti,ab OR transients:ti,ab OR migrant:ti,ab OR migrants:ti,ab
Children	'child'/de OR 'pediatrics'/de OR 'adolescent'/de	Child*:ti,ab OR pediatric*:ti,ab OR adolescen*:ti,ab OR ‘young adult’:ti,ab OR youth:ti,ab
ADHD	'attention deficit disorder'/de OR 'hyperkinesia'/de	‘Attention Deficit Disorder’:ti,ab OR ADHD:ti,ab OR ‘Attention Deficit Disorder with Hyperactivity’:ti,ab OR ADD:ti,ab OR hyperactiv*:ti,ab OR hyperkinesis:ti,ab OR inattention:ti,ab OR impulsiv*:ti,ab OR overactiv*:ti,ab OR externaliz*:ti,ab
Meditation	'meditation'/exp OR 'mindfulness'/de	Meditat*:ti,ab OR mindful*:ti,ab OR transcendental:ti,ab OR vipassana:ti,ab OR ‘loving kindness’:ti,ab OR ‘mindful-self compassion’:ti,ab OR yoga:ti,ab OR zen:ti,ab OR Sudarshan:ti,ab OR ‘chi kung’:ti,ab OR qigong:ti,ab OR kirtan:ti,ab OR pranayama:ti,ab OR ‘acceptance commitment therapy’:ti,ab
Results	Date limit: 2012-2023<br>Results: 31<br>Searched on 12/30/23	
**3. PsycINFO**		

Concept	Controlled Vocab	Keywords
Minority	MAINSUBJECT.EXACT("Marginalized Groups") OR MAINSUBJECT.EXACT("Minority Groups") OR MAINSUBJECT.EXACT("Health Disparities")	underserved OR minority OR minorities OR homeless* OR impaired OR uninsured OR underinsured OR african* OR black OR blacks OR "native american" OR "native americans" OR gypsy OR gypsies OR hispanic OR hispanics OR latino OR latinos OR latina OR latinas OR chicano OR chicanos OR "mexican american" OR "mexican americans" OR mexican-american* OR "puerto rican" OR "puerto ricans" OR jamaican OR jamaicans OR immigrant OR immigrants OR refugee OR refugees OR transients OR migrant OR migrants
Children	MAINSUBJECT.EXACT("Adolescent Health") OR MAINSUBJECT.EXACT("Pediatrics")	Child* OR pediatric* OR adolescen* OR "young adult" OR youth
ADHD	MAINSUBJECT.EXACT("Attention Deficit Disorder with Hyperactivity") OR MAINSUBJECT.EXACT("Hyperkinesis") OR MAINSUBJECT.EXACT("Attention Deficit Disorder")	"Attention Deficit Disorder" OR ADHD OR "Attention Deficit Disorder with Hyperactivity" OR ADD OR hyperactiv* OR hyperkinesis OR inattention OR impulsiv* OR overactiv* OR externaliz*
Meditation	MAINSUBJECT.EXACT("Mindfulness") OR MAINSUBJECT.EXACT("Meditation") OR MAINSUBJECT.EXACT("Mindfulness-Based Interventions")	Meditat* OR mindful* OR transcendental OR vipassana OR "loving kindness" OR "mindful-self compassion" OR yoga OR zen OR sudarshan OR "chi kung" OR qigong OR kirtan OR pranayama OR "acceptance commitment therapy"
Results	Date limit: 1/1/2012- 12/30/2023<br>Results: 33<br>Searched on 12/30/23	

4. Web of Science

Concept	Controlled Vocab	Keywords
	N/A	"Health Disparity, Minority and Vulnerable Populations" OR "Minority Health" OR "Minority Groups" OR "Ethnic and Racial Minorities" OR underserved OR minority OR minorities OR homeless* OR impaired OR uninsured OR underinsured OR african* OR black OR blacks OR "native american" OR "native americans" OR gypsy OR gypsies OR hispanic OR hispanics OR latino OR latinos OR latina OR latinas OR chicano OR chicanos OR "mexican American" OR "mexican americans" OR mexican-american* OR "puerto rican" OR "puerto ricans" OR jamaican OR jamaicans OR immigrant OR immigrants OR refugee OR refugees OR transients OR migrant OR migrants
Children	N/A	Child* OR pediatric* OR adolescen* OR "young adult" OR youth
ADHD	N/A	"Attention Deficit Disorder" OR ADHD OR "Attention Deficit Disorder with Hyperactivity" OR ADD OR hyperactiv* OR hyperkinesis OR inattention OR impulsiv* OR overactiv* OR externaliz*
Meditation	N/A	Meditat* OR mindful* OR transcendental OR vipassana OR "loving kindness" OR "mindful-self compassion" OR yoga OR zen OR sudarshan OR "chi kung" OR qigong OR kirtan OR pranayama OR "acceptance commitment therapy"
Results	Publication years: 01/01/2012-12/31/2023<br>Results: 30<br>Searched on 12/30/23	

5. Cochrane Reviews

Reviews: 0
Trials: 15
Searched on 12/30/23

ID	Search	Hits
#1	MeSH descriptor: [Health Disparity, Minority and Vulnerable Populations] explode all trees	2962
#2	MeSH descriptor: [Minority Health] this term only	25
#3	MeSH descriptor: [Minority Groups] this term only	389
#4	MeSH descriptor: [Ethnic and Racial Minorities] this term only	6
#5	(underserved OR minority OR minorities OR homeless* OR impaired OR uninsured OR underinsured OR african* OR black OR blacks OR "native american" OR "native americans" OR gypsy OR gypsies OR hispanic or hispanics OR latino OR latinos OR latina OR latinas OR chicano OR chicanos OR "mexican american" OR "mexican americans" OR "puerto rican" OR "puerto ricans" OR jamaican OR jamaicans OR immigrant or immigrants OR refugee OR refugees OR transients OR migrant OR migrants):ti,ab,kw	66859
#6	MeSH descriptor: [Child] this term only	52455
#7	MeSH descriptor: [Pediatrics] this term only	667
#8	MeSH descriptor: [Adolescent] this term only	109121
#9	(Child* OR pediatric* OR adolescen* OR "young adult" OR youth):ti,ab,kw	321648
#10	MeSH descriptor: [Attention Deficit Disorder with Hyperactivity] this term only	2976
#11	MeSH descriptor: [Attention Deficit and Disruptive Behavior Disorders] this term only	325

#12 MeSH descriptor: [Attention Deficit Disorder with Hyperactivity] this term only and with qualifier(s): [therapy - TH] 521
#13 MeSH descriptor: [Hyperkinesis] this term only 617
#14 (“Attention Deficit Disorder” OR ADHD OR “Attention Deficit Disorder with Hyperactivity” OR ADD OR hyperactiv* OR hyperkinesis OR inattention OR impulsiv* OR overactiv* OR externaliz*):ti,ab,kw 30008
#15 MeSH descriptor: [Mindfulness] this term only 1174
#16 MeSH descriptor: [Meditation] this term only 696
#17 (Meditat* OR mindful* OR transcendental OR vipassana OR “loving kindness” OR “mindful-self compassion” OR yoga OR zen OR sudarshan OR “chi kung” OR qigong OR kirtan OR pranayama OR “acceptance commitment therapy”):ti,ab,kw 12180
#18 #1 OR #2 OR #3 OR #4 OR #5 67236
#19 #6 OR #7 OR #8 OR #9 321648
#20 #10 OR #11 OR #12 OR #13 OR #14 30125
#21 #15 OR #16 OR #17 12180
#22 #18 AND #19 AND #20 AND #21 15
